# Tropical Forest Soil Microbiome Modulates Leaf Heat Tolerance More Strongly Under Warming Than Ambient Conditions

**DOI:** 10.1002/ece3.71425

**Published:** 2025-05-14

**Authors:** Gabriela Hernandes Villani, Iana F. Grullón‐Penkova, Parker Bartz, Joel Masanga, Jesse R. Lasky, Molly A. Cavaleri, Tana E. Wood, Benedicte Bachelot

**Affiliations:** ^1^ Department of Plant Biology Ecology and Evolution Oklahoma State University Stillwater Oklahoma USA; ^2^ USDA Forest Service International Institute of Tropical Forestry Río Piedras Puerto Rico USA; ^3^ Department of Biology Pennsylvania State University University Park Pennsylvania USA; ^4^ Michigan Technological University Houghton Michigan USA

**Keywords:** climate change, global warming, heat stress, leaf heat tolerance, microbiome, tropical ecology, tropical forest

## Abstract

It is unclear how plants respond to increasing temperatures. Leaf heat tolerance (LHT) is often at its upper limit in tropical forests, suggesting that climate change might negatively impact these forests. We hypothesized that intraspecific variation in LHT might be associated with changes in the soil microbiome, which might also respond to climate. We hypothesized that warming would increase LHT through changes in the soil microbiome: we combined an in situ tropical warming experiment with a shade house experiment in Puerto Rico. The shade house experiment consisted of growing seedlings of *Guarea guidonia* , a dominant forest species, under different soil microbiome treatments (reduced arbuscular mycorrhizal fungi, reduced plant pathogens, reduced microbes, and unaltered) and soil inoculum from the field experiment. Heat tolerance was determined using chlorophyll fluorescence (*F*_*V*_ */F*_*m*_) on individual seedlings in the field and on groups of seedlings (per pot) in the shade house. We sequenced soil fungal DNA to analyze the impacts of the treatments on the soil microbiome. In the field, seedlings from ambient temperature plots showed higher *F*_*V*_ */F*_*m*_ values under high temperatures (0.648 at 46°C and 0.067 at 52°C) than seedlings from the warming plots (0.535 at 46°C and 0.031 at 52°C). In the shade house, the soil microbiome treatments significantly influenced the fungal community composition and LHT (*T*_*crit*_ and *F*_*V*_ */F*_*m*_). Reduction in fungal pathogen abundance and diversity altered *F*_*V*_ */F*_*m*_ before *T*_50_ for seedlings grown with soil inoculum from the warming plots but after *T*_50_ for seedlings grown with soil inoculum from the ambient plots. Our findings emphasize that the soil microbiome plays an important role in modulating the impacts of climate change on plants. Understanding and harnessing this relationship might be vital for mitigating the effects of warming on forests, emphasizing the need for further research on microbial responses to climate change.

## Graphical abstract

Leaf heat tolerance (LHT) is often at its upper limit in tropical forests, suggesting that climate change might negatively impact these forests. We found that intraspecific variation in this leaf trait was associated with changes in the soil microbiome and its responses to climate.

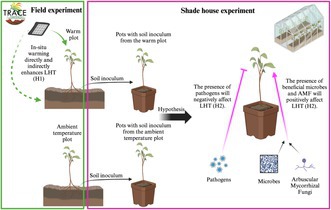

## 1 Introduction

During the last century, the global temperature has increased by 1°C, and climate models predict that it will increase by 3.5°C–4.0°C by the end of the century (Parry et al. 2007), which is a possible turning point in plant metabolic function (Doughty et al. 2023). Leaf temperature is altered by sunlight, wind, and water availability and usually exceeds air temperature during periods of intense sunlight (Matsui and Eguchi 1971; Perera et al. 2019) by up to 10°C in tropical forests (Miller et al. 2021; Crous et al. 2023). Tropical plants tend to be at their maximal temperature tolerance (Tiwari et al. 2021; Sentinella et al. 2020) and photosynthetic thermal optima (Mau et al. 2018). Moreover, the low acclimation capacity of many tropical plants is inadequate to stay within their narrow leaf thermal safety margins, which is the difference between photosynthetic thermal tolerance and maximum leaf temperature at higher growth temperatures, making them more susceptible to increased thermal stress (Tarvainen et al. 2022; Kullberg et al. 2024). There is an increasing need to understand the factors that modulate plant responses to warming temperature, especially in tropical forests.

Leaf heat tolerance (LHT) is a functional trait describing the ability of a leaf to withstand high temperatures. This trait is determined by maximum quantum efficiency of the protein‐complex Photosystem II (*F*_*V*_ */F*_*m*_; Baker and Rosenqvist 2004; Krause et al. 2013), which is the ratio of maximum variable to maximum total fluorescence (Krause et al. 2010). As the leaf experiences high temperatures, *F*_*V*_ */F*_*m*_ declines. Leaf heat tolerance is often defined by the parameters best describing this decline in maximum quantum efficiency of protein‐complex Photosystem II, *T*_50_, and *T*_*crit*_. *T*_50_ is the temperature where the efficiency of the protein‐complex Photosystem II is reduced by 50% whereas *T*_*crit*_ is the critical temperature at which leaves start to exhibit damage (Perez and Feeley 2020; Slot et al. 2021). High LHT is associated with high *T*_50_ and *T*_*crit*_. In contrast, a low LHT corresponds to a low *T*_50_ and *T*_*crit*_. Understanding variations in *F*_*V*_ */F*_*m*_ and in these two commonly used LHT parameters may help predict how tropical forests respond to a warming climate.

Leaf heat tolerance exhibits high intraspecific variation caused by abiotic and biotic factors (Teskey et al. 2015). Abiotic factors, such as prior exposure to elevated temperatures, result in heat stress priming and memory, which can enhance plant resilience to subsequent episodes of thermal stress (Bäurle 2016; Friedrich et al. 2019). Priming is an example of acclimation (Filaček et al. 2022), by which the plants respond to prior elevated temperatures by activating heat shock transcription factors, boosting their response to future elevated temperatures (Bäurle 2016). Heat stress memory happens when exposure to prior elevated temperatures leads to changes in the chromatin structure, which could result in future heat tolerance for the plants and their offspring (Bäurle 2016).

Besides abiotic factors, plant tolerance to heat stress can be mediated by biota such as beneficial fungi, which can improve plants' water uptake (Hubbard et al. 2014), or other beneficial soil microbes, which can boost plants' responses to heat stress (Shekhawat et al. 2022; Parasar et al. 2024). Thus, we hypothesize that associations between plants with beneficial bacteria or fungi increase LHT. Plant response to stress includes modifications in the levels, types, and regulation of primary and secondary plant metabolites (Ramakrishna and Ravishankar 2011). Amino acids and sugars are primary plant metabolites vital for plant vitality and growth; alkaloids and flavonoids are secondary metabolites known to serve defensive purposes (Ramakrishna and Ravishankar 2011). Due to the costs of abiotic stress response, plants developed a delicate equilibrium between allocating resources to stress management versus growth and reproduction (Abd El‐Daim et al. 2019). Yet, plant performance under heat stress tends to increase if the plants engage in beneficial associations (Abd El‐Daim et al. 2019; Waqas et al. 2015). Associations with beneficial symbionts help the plants allocate more resources toward both primary and secondary compounds, thereby supporting both growth and stress tolerance (Abd El‐Daim et al. 2019). In particular, the use of microbial inoculum in crops has highlighted several direct (e.g., phytohormonal regulation such as induction of stress responsive pathways leading to a reduction in the unsaturated levels of fatty acid in the cell membrane) and indirect (e.g., induction of systemic acquired resistant or production of antioxidant reducing chloroplast and membrane injury) mechanisms by which beneficial microbes can help plants alleviate heat stress (Sarkar et al. 2018; Rawat et al. 2021; Jyoti Parasar et al. 2024). As a result, plants associating with beneficial bacteria and fungi might exhibit higher LHT, characterized by high *F*_*V*_ */F*_*m*_, *T*_50_, and *T*_*crit*_. Among the beneficial fungi, arbuscular mycorrhizal fungi (AMF) are important in tropical forests, as they form a symbiotic relationship with more than 80% of tropical plant species (Tedersoo et al. 2018). Studies have highlighted the role of AMF in plant nutrient uptake, including calcium (Sardans et al. 2023) which is associated with high LHT (Jiang and Huang 2001). Therefore, we hypothesize that AMF might be an important player in the soil microbiome with the potential to enhance LHT.

In contrast, fungal pathogens might be associated with low LHT, characterized by low *F*_*V*_ */F*_*m*_, *T*_50_, and *T*_*crit*_. Plant–pathogen interactions can be influenced by season, host plant, and environment (Elad and Pertot 2014; Velásquez et al. 2018), as well as the combined impact of these interactions (Nelson 1994). An interaction with pathogens triggers defense pathways mediated by phytohormones, protein kinases, and programmed cell death in plants (Suzuki and Katano 2018). As a result, the plant's ability to fight off pathogens may drain resources needed to maintain high levels of LHT. Therefore, we hypothesize that pathogens should be associated with low LHT. Alternatively, because of similarities in the signaling pathways involved in the response to pathogen attack and heat stress, one could expect higher LHT in plants interacting with pathogens than in healthy plants (Suzuki and Katano 2018).

The soil microbiome may also be directly affected by climate change (Cao et al. 2020; Nottingham et al. 2022). For example, in an experiment warming soil to +5°C, AMF growth rates were increased for a few months, followed by a reduction in AMF diversity and a change in AMF community composition as the warming continued for 3 years (Cao et al. 2020). Similarly, bacterial diversity increased and peaked quickly before decreasing in a 2‐year soil warming experiment in Panama (Nottingham et al. 2022). Finally, the life cycle of pathogens is influenced by temperature, which alters their survival and multiplication rates (Bale et al. 2002). High temperatures can boost or reduce the receptiveness of plants to a pathogen, depending on the host and the capacity of the pathogen to tolerate heat (Bale et al. 2002). Thus, climate change can alter the soil biota composition, which might influence plant LHT.

**FIGURE 1:**
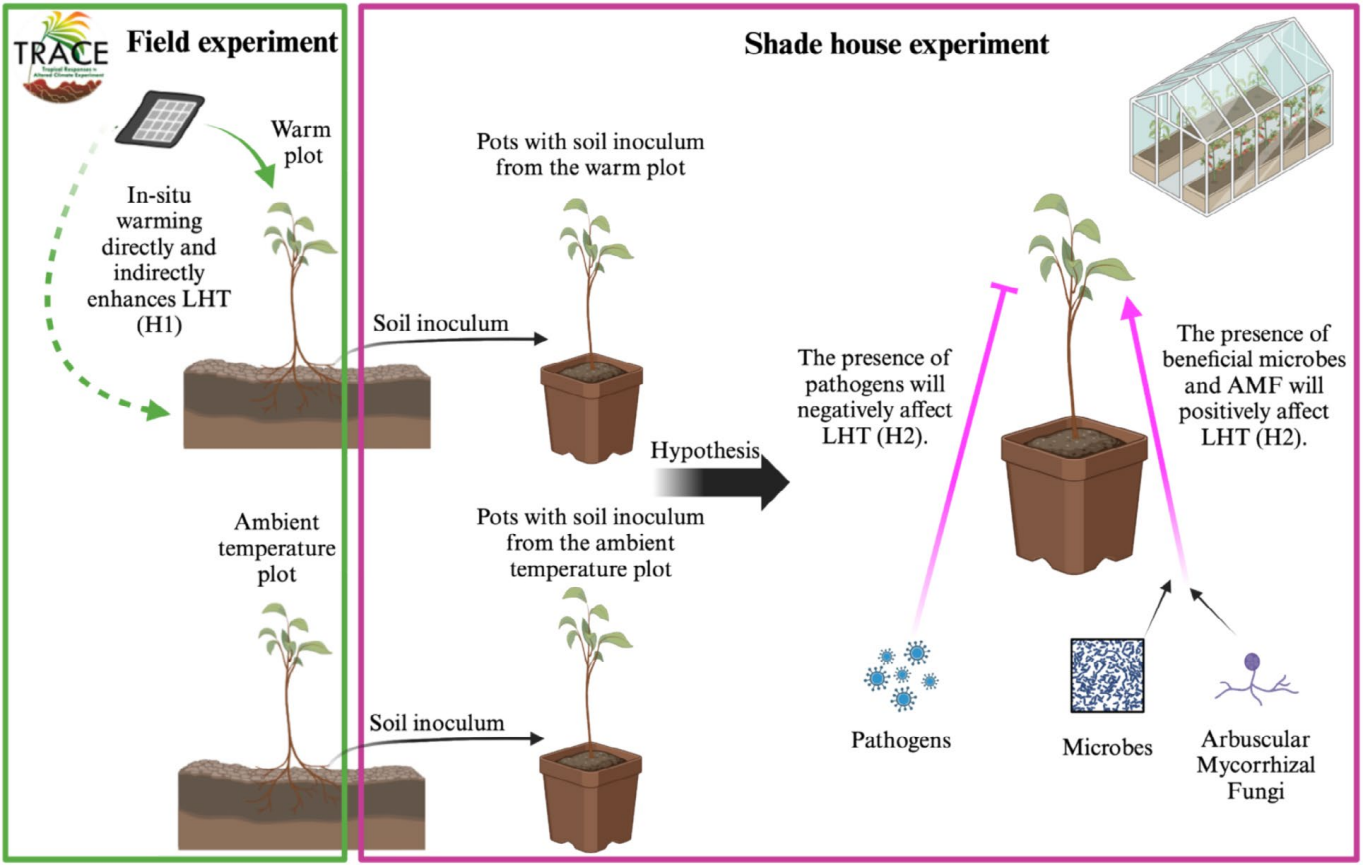
Graphical representation of hypotheses in the present study. Under favored hypotheses, experimental warming underlies variation in leaf heat tolerance (LHT) by directly (plain line) impacting the plants and indirectly (dotted line) by regulating the microbiome under field (green: H1) and shade house (purple: H2) experimental conditions. Arrows represent predicted positive effects on LHT whereas perpendicular lines represent hypothesized negative effects on LHT.

At the Tropical Responses to Altered Climate Experiment (TRACE) in Puerto Rico, early analyses of understory responses to warming highlighted interesting changes in biotic interactions due to altered climate (Bachelot et al. 2020). Seedling survival was increased in a +4°C warming treatment when surrounded by high conspecific density without a decrease in aboveground natural enemies, suggesting potential long‐term impacts on tropical wet forest diversity under future climate conditions (Bachelot et al. 2020; Alonso‐Rodríguez et al. 2022). In the present study, we used TRACE and a shade house experiment, and we hypothesized that experimental warming regulates LHT by influencing the soil microbiome (Figure 1). Under this hypothesis, we predicted that (i) LHT would be higher in the warmed plots compared to ambient due in part to inherent differences in the microbial composition; and (ii) high relative abundances of fungal pathogens and beneficial communities like AMF would decrease and increase LTH, respectively. We tested this hypothesis using field and shade‐house experiments.

## 2 Methodology

### 2.1 Site and TRACE Field Experiment

This study was conducted at the USDA Forest Service Sabana Field Research Station in Luquillo, northeastern Puerto Rico (18°18′ N, 65°50′ W). The area is described as a subtropical wet forest (Holdridge 1967), typically aseasonal (Bouskill et al. 2016). It is a secondary forest that has naturally been recovering from pasture for almost 73 years (Kimball et al. 2018). The forest mainly comprises *Prestoea montana, Psychotria brachiata, Syzygium jambos*, and *Sloanea berteriana* (Cook et al. 2013). Rainfall is at least 200 mm per month and varies significantly throughout the year (Heartsill‐Scalley et al. 2007), while the average annual temperature is 24°C with low variation between months (4°C, Kimball et al. 2018). The soil is described as Ultisol, with an elevated amount of weathered clay (Scatena 1989).

The TRACE field experiment was established in 2015 to investigate understory plant and soil responses to +4°C warming, using six 12 m^2^ plots (Kimball et al. 2018; Tunison et al. 2024). Three of these plots are heated using infrared heaters at 4°C above the ambient level, suspended approximately 3.6 m above the ground on crossbars. In contrast, the soil temperature sensors indicate that the soil in the warmed plots was about 2°C higher than the soil temperature in the ambient temperature plots at the time of the study (Wood et al. 2024). The remaining three serve as control plots, equipped with identical infrastructure but utilizing metal plates instead of heaters to simulate the physical presence of heating equipment without altering temperature. These plots are spaced about 10 m apart in an open area without mature trees to accommodate the experimental setup (Bachelot et al. 2020; Alonso‐Rodríguez et al. 2022; Kimball et al. 2018; Reed et al. 2020). The study's warming treatment ceased on Sept 6, 2017, after 11.5 months, coinciding with Hurricane Irma's approach 97 km north of Puerto Rico at Category 5 strength, followed by Hurricane María crossing the island on Sept 20, 2017, as a Category 4 storm (Yaffar et al. 2021). Warming was initiated again in September 2018 (Alonso‐Rodríguez et al. 2022), being a 5‐year warming experiment by the time of the data collection for this study which happened in July 2023.

### 2.2 Field Data Collection

To assess the impact of increased temperatures on leaf heat tolerance (LHT), we focused on the dominant forest species, *Guarea guidonia* . This choice was informed by the species' abundant seed supply and the availability of previous LHT data from earlier studies at the TRACE location (Carter and Cavaleri 2018; Carter et al. 2020). *Guarea guidonia* , commonly known as Muskwood, is an evergreen tree that can reach up to 23 m in height, with a straight trunk up to 90 cm in diameter. *Guarea guidonia* is a late‐successional broadleaved evergreen (Pennington and Clarkson 2013).

In July 2023, we collected data from fully developed top leaves from two *Guarea guidonia* seedlings available per plot (three ambient and three warmed plots), which have been under heat treatment since their germination. We chose seedlings with similar heights (mean = 20.31 cm, range: 13–45 cm) and diameters (mean = 3.65 mm, range: 2.2–9.5 mm), and measured the height and diameter of each individual. We used a disk paper puncher (0.635 cm, Kawendite) to collect 18 leaf disks per plant and stored them in Ziploc bags for LHT assessment in the laboratory (see the Section 2.4). Finally, with a sterilized metal spoon, we collected one soil sample from a soil core from each plot and kept it in sterile plastic bags in a −80°C freezer until further analysis (see soil DNA analyses).

### 2.3 Shade House Experiment

**FIGURE 2:**
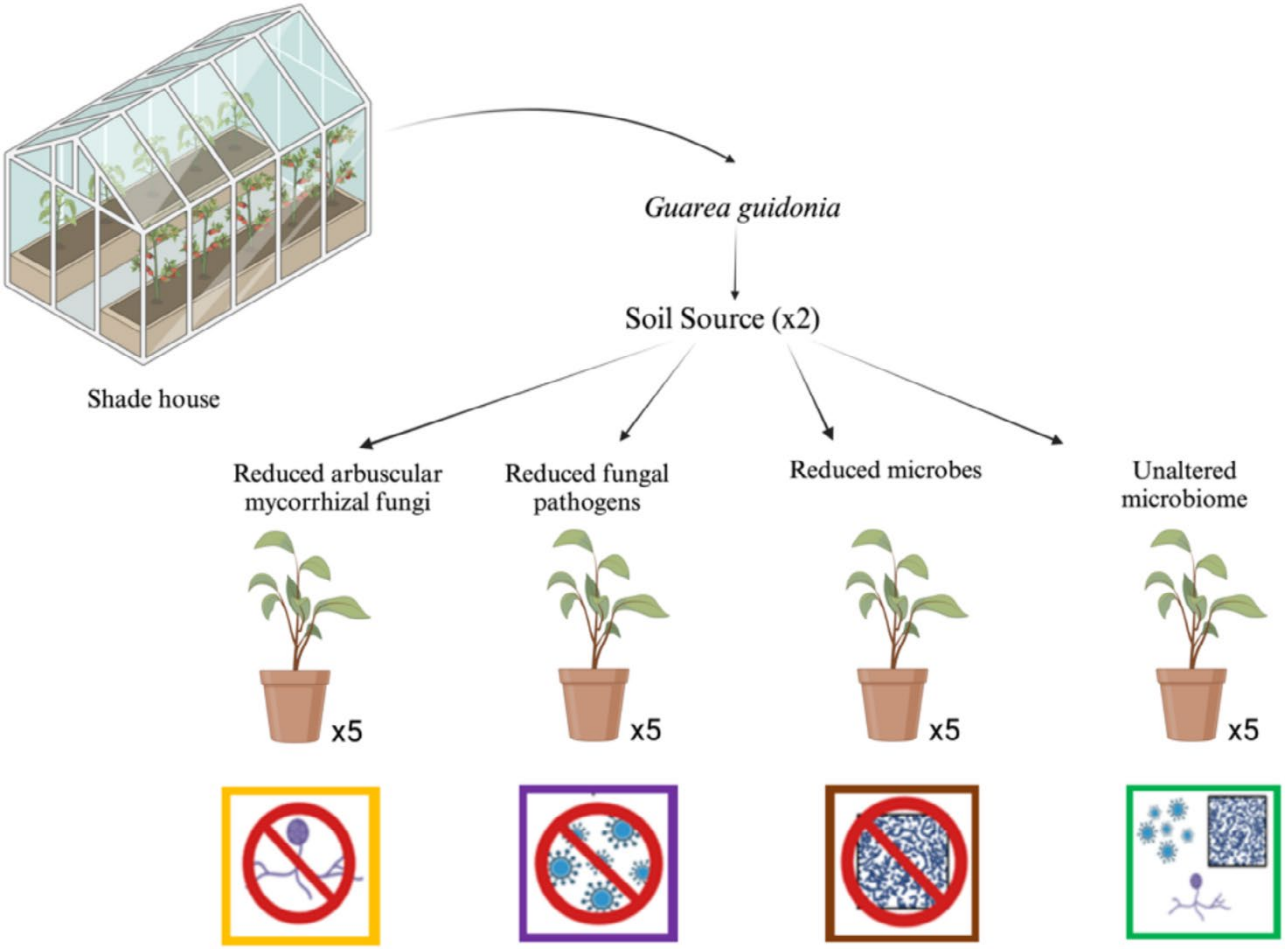
Schematic of the experiment. We grew *Guarea guidonia* in pots of five seedlings each with two soil sources for inoculum: Soil inoculum from the warmed plots and the ambient temperature plots. We exposed them to four different treatments replicated five times: Reduced arbuscular mycorrhizal fungi (AMF) (with microbes and fungal pathogens, in yellow), reduced fungal pathogens (with microbes and AMF, in purple), reduced microbes (with AMF and fungal pathogens, in brown), and unaltered microbiome, in green. Created with BioRender.com.

We used a fully factorial design where 240 seedling pots were randomly assigned to one of four soil microbiome treatments with soil inoculum from either warmed or ambient plots. Pots were placed across four blocks, each assigned to low or high soil moisture treatment. For this study, we measured data on seedlings from the high‐density treatment (to ensure enough plant material) and high soil moisture treatment to avoid confounding microbiome treatment and water stress effects of LHT. Each combination of treatments (soil inoculum × microbiome × seedling density × soil moisture) was replicated five times. Briefly, *G. guidonia* seeds were collected in January 2023 from around the Sabana Field Research Station, and their surface was sterilized using 5% sodium hypochlorite for 5 min as previously described (Sauer and Burroughs 1986). The seeds were pre‐germinated in sterile soil and transplanted on March 13th 2023 (1, 3, and 5 seeds for low, medium, and high density, respectively) in pots with 250 g of oven‐sterilized soil primed with 5 g of inoculum from the ambient or warmed plots (Figure 2). The four soil treatments were: unaltered microbiome, reduced AMF, reduced soil fungal pathogens, and reduced microbes. Reduced AMF was achieved by diluting 3.25 g of Banrot 40wp fungicide ((3‐(2‐ methyl piperidine)‐propyl‐3,4‐dichloro benzoate) and 3.6 L of deionized (DI) water. Banrot is known for managing damping‐off, root, and stem rot diseases instigated by *Pythium, Phytophthora, Rhizoctonia, Fusarium*, and *Thielaviopsis* (Prabhakaran and Dann 2022). More importantly, Banrot is similar to Topsin‐M, which was found to significantly reduce AMF in the field (Wilson and Williamson 2008). Subsequently, 60 mL of the diluted Banrot solution was poured into each pot and randomly assigned to the treatment. Reduced fungal pathogens were obtained by mixing 0.185 mL of Abound Flowable fungicide (Azoxystrobin: methyl(E)‐2‐{2‐[6‐(2‐cyanophenoxy) pyrimidin‐4‐yloxy] phenyl}‐3‐methoxyacrylate) with 600 mL of DI water. Abound is a fungicide used for comprehensive disease management (Starkey et al. 2013). 2 mL of the diluted Abound solution was sprayed onto seedlings' leaves in each pot assigned to this treatment. Finally, for the reduced microbe treatment, the soil inoculum was oven‐dried for 48 h at 70°C before pot filling. Both fungicide treatments (Banrot and Abound) were reapplied to respective treatments once every month.

Seedlings in the shade house experienced low light conditions (shade was 60% and shade house was positioned in the shade of a building) and average temperature of 26°C ± 3°C, which was similar to field conditions. Seedlings were allowed to grow for 3 months before LHT measurement. Three pots with the most leaf material available were randomly selected from each of the five‐pot replicates per treatment to collect leaf disks in July 2023. Each seedling from which leaf disks were collected was measured for height (mean = 11.55 cm, range: 5.1–18.5 cm) and diameter (mean = 3.32 mm, range: 1.9–3.8 mm). Using an 0.635 cm disk paper puncher (Kawendite), 18 leaf disks per selected pot were randomly collected, placed in a plastic bag, and immediately taken to the laboratory to measure LHT (see Figure S1). We combined the 18 leaf disks from one pot into one measurement of LHT because individual seedlings did not have enough leaf material. Therefore, the shade house data is at the seedling pot level. Finally, we collected soil samples from each pot at the end of the shade house experiment and kept them in sterile bags in a −80°C freezer until further analyses (see soil DNA analyses).

### 2.4 Leaf Heat Tolerance Measurement

Leaf disks were exposed to heat stress to measure LHT, following a method adapted from Krause et al. (2010). Leaf disks from both field and shade house experiments were collected early in the morning and immediately taken to the laboratory protected in a cloth inside Ziploc bags for LHT measurement. Approximately 30 min after collection, samples were ready to be incubated for 15 min in temperature‐controlled water baths (water heater model ANOVA Sous vide 2.0, 3.0, precision +/−0.2°C) set at one of five temperatures, targeted as a common range for *T*_50_ occurrence (Krause et al. 2010, 2013): 46°C, 48°C, 50°C, 52°C, and 54°C. This temperature range was first decided after testing two different starting temperatures (44°C vs. 46°C) with extra leaf samples (Perez and Feeley 2020; Slot et al. 2021). In addition, a sample was set at ambient temperature in the laboratory (25°C) for control. 24 h later, leaf disks were placed in the dark for 15 min before *F*_*V*_ */F*_*m*_ was measured using a fluorometer (model OS30p from Opt‐Sciences). For each sample (individual plants in the field or pots in the shade house), *F*_*V*_ */F*_*m*_ was measured using three leaf disks per temperature, totaling 18 leaf disks per sample.

*F*_*V*_ */F*_*m*_ data were used to calculate several key parameters (*T*_50_ and *T*_*crit*_) for each sample. *T*_50_ is the temperature at which the potential quantum efficiency of Photosystem II (*F*_*V*_ */F*_*m*_) is reduced by 50% (Krause et al. 2010). *F*_*V*_ */F*_*m*_ values after 24 h of heat treatment is the parameter most closely associated with permanent leaf damage (dark coloration and necrosis, Krause et al. 2010). The parameter *b* represents the steepness of the decrease in *F*_*V*_ */F*_*m*_ slope at *T*_50_. These parameters were obtained by fitting the *F*_*V*_ */F*_*m*_ data to the following equation:

$$F_{V}/F_{m}=\frac{F_{V}/F_{m,\max}}{1+e^{b\left(T_{\text{leaf}}-T_{50}\right)}}$$

In this equation, *F*_*V*_ */F*_*m*_,_max_ is the upper horizontal asymptote representing *F*_*V*_ */F*_*m*_ associated with healthy, nonstressed leaves. Finally, *T*_*leaf*_ is the leaf disk's incubation temperature (in °C). Once *T*_50_ and *b* are estimated, the *T*_*crit*_ parameter is obtained as the temperature where the line describing the slope of *F*_*V*_ */F*_*m*_ declines at *T*_50_ (*b*) intersects with the asymptotic line defined by *F*_*V*_ */F*_*m*_,_*max*_.

### 2.5 Soil DNA Extraction and Sequencing

To characterize fungal community composition, we extracted and sequenced DNA from soil samples collected from warmed and control field plots as well as pots in the shade house. Briefly, a soil sample was scooped using a sterilized spoon at the center of each pot in the shade house and cores from the field, which were frozen at −80°C in sterilized bags until later, to be subjected to DNA extraction using the PowerSoil Pro Kit (Qiagen, Germantown, MD, USA) according to the manufacturer's instructions. Subsequently, the DNA extractions were sequenced via Illumina sequencing at the University of Minnesota Genomic Center (UMGC), using fungal‐specific primers targeting the internal transcribed spacer 2 region (ITS4_Nextera and JL0015.8SR_Nextera). The University of Minnesota Genomic Center protocol includes a 3 min 95°C denaturation phase followed by a 20 s 98°C phase to activate the DNA polymerase, a 15 s 65.7°C annealing phase, and a 45 s 72°C elongation phase for 30 cycles, followed by a final 5 min 72°C elongation (Gohl et al. 2016). To ensure data quality and correct sample differentiation, UMGC conducted preliminary quality control and demultiplexing. Sequencing data were analyzed using the Pete supercomputer at Oklahoma State University and the Mothur pipeline (Schloss et al. 2009). After detecting and removing chimeras, this pipeline generates operational taxonomical units (OTUs) by grouping sequences at 97% identity (Gweon et al. 2015) using the UNITE v9 database (Abarenkov et al. 2024). The resulting OTUs were taxonomically classified into phylotypes and putative guilds utilizing FUNGuild, a Python‐based tool designed for ecological interpreting of fungal OTUs (Nguyen et al. 2016).

### 2.6 Statistical Analyses

To test whether warming influenced leaf heat tolerance in *G. guidonia* in the field (H1), we first tested how the maximum quantum efficiency of PSII (*F*_*V*_ */F*_*m*_) differed between the ambient and warmed plots at each temperature (Slot et al. 2019), using a Welch's *t*‐test. We also fitted mixed linear regressions to explain each estimated leaf heat tolerance parameter (*T*_50_ and *T*_*crit*_) as a function of warming treatment and seedling height (actual size for the field data, or averaged within a pot in the shade house) as fixed effects and plots as a random effect.

To test whether the soil microbiome influenced leaf heat tolerance in the shade house (H2), we first investigated how *F*_*V*_ */F*_*m*_ differed across biotic treatments at each temperature (Slot et al. 2019) using an analysis of variance (one‐way ANOVA) and Tukey post hoc test to analyze which pairwise treatment differences were significant. This analysis was conducted within soil source. We then fitted a linear regression to explain each estimated leaf heat tolerance parameter (*T*_50_ and *T*_*crit*_) as a function of seedling average height, number of seedlings alive in the pot, microbiome treatment, soil source, and the interactions between soil source and microbiome treatment as fixed effects. Leaf heat tolerance parameters were obtained for a pot and not for an individual seedling (not enough leaf material), so we used the average seedling height as a covariate since leaf heat tolerance might change along ontogeny (Klockmann et al. 2017). We used the uncertainty (standard error) from the fitted leaf heat tolerance parameters for each model as weights to account for errors in the response variable (heat tolerance parameters). Finally, we calculated estimated marginal means using the R package *emmeans* (Lenth 2022) for pairwise comparison across treatments and soil source in the shade house.

To test for differences in fungal composition, we used redundancy analyses to test whether in situ warming and soil microbiome treatments influenced the fungal community composition after verifying that fungal composition had homogeneous variances across plots and soil microbiome treatments. Furthermore, to identify how fungal classes differ among the treatments, we conducted an ANOVA followed by a Tukey post hoc test to assess which fungal classes' relative abundance differs across soil microbiome treatments.

All the analyses were conducted in R (version 4.2.1; R Core Team 2022), using *nls.multstart* (Padfield and Matheson 2018), *lmerTest* (Kuznetsova et al. 2017), and *vegan* (Oksanen 2022) packages.

## 3 Results

### 3.1 Results From the Field

#### 3.1.1 Warming Lowers *F*_*V*_ */F*_*m*_ in the Field

**FIGURE 3:**
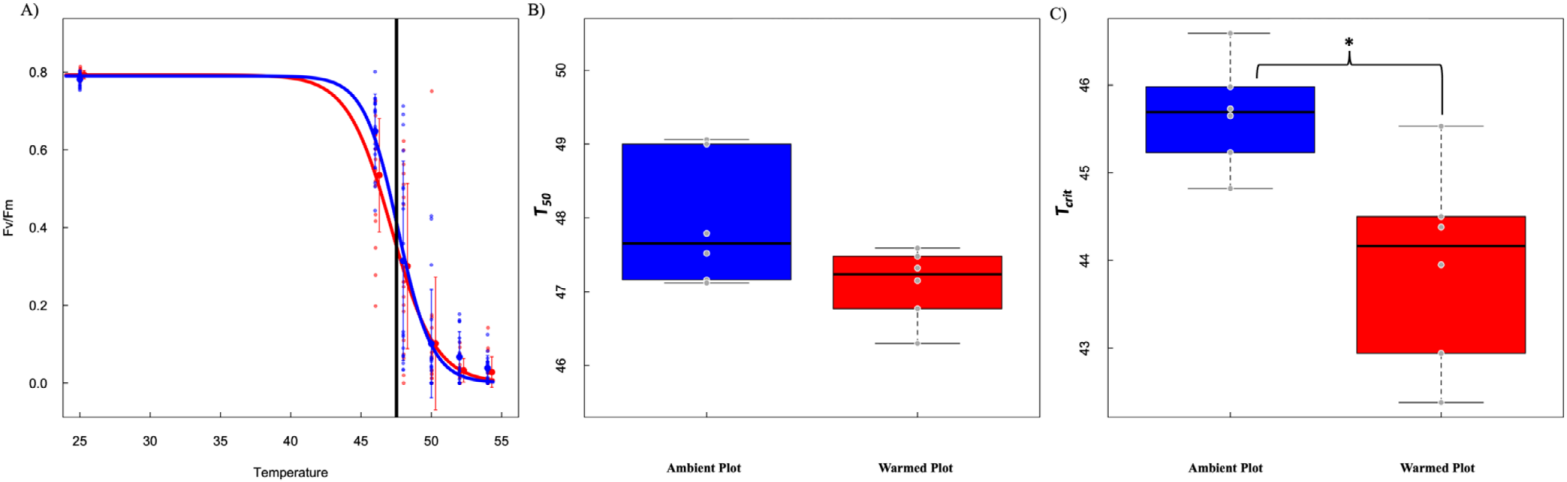
(A) Ratio of maximum variable to maximum total fluorescence (*F*_*V*_/*F*_*m*_) values according to the soil source from the plots (ambient (blue line) and warmed (red line)). The large dots and vertical bars show the *F*_*V*_/*F*_*m*_ mean and 95% quantiles at each temperature. The small dots represent the data points (*n* = 18 = 3 disks × 2 individuals × 3 plots at each temperature for each treatment). The black vertical line represents the mean *T*_50_ of seedlings in ambient and warmed plots. (B) and (C) are boxplots of the *T*_50_ and *T*_*crit*_ parameters in ambient and warmed plots (*n* = 6 in each treatment).

As expected, *F*_*V*_ */F*_*m*_ (Figure 3) was lower in seedlings from the warmed plots compared to ambient plots with mean values of *F*_*V*_ */F*_*m*_ of 0.535 in the warmed plot and 0.648 in the ambient temperature plot for a 46°C heat stress (*t*_29_ = 2.74, *p* = 0.01) and 0.067 in the ambient temperature plot and 0.031 in the warmed plot for a 52°C heat stress (*t*_24_ = 2.04, *p* = 0.05). This result was reversed at ambient temperature (25°C), where *F*_*V*_ */F*_*m*_ (Figure 3) was higher in seedlings from the warmed plots (*t*_30_ = −2.69, *p* = 0.011) with mean *F*_*V*_ */F*_*m*_ values of 0.792 compared to seedlings from the ambient temperature plots with mean *F*_*V*_ */F*_*m*_ values of 0.781.

Contrary to expectations, there was no significant difference in *T*_50_ between seedlings grown in warmed versus ambient plots, but *T*_*crit*_ was significantly lower in the warmed plots compared with ambient plots (*F*_1,9_ = 11.45, *p* < 0.001, Figure 3B,C).

#### 3.1.2 Warming Partially Influences Microbiome Composition in the Field

We hypothesized that the warming treatment might alter the microbial community structure. The analyses of the soil inoculum from the plots showed no significant effect of warming on overall fungal community composition, using the ANOVA test. The relative abundance of fungal classes most represented by pathogens such as *Dothideomycetes*, *Eurotiomycetes*, and *Sordariomycetes* was not significantly different between warmed and ambient plots. However, *Glomeromycetes*, which is an AMF class, is most present in the inoculum from the ambient plots, and *Archaeosporomycetes*, another AMF class, is just present in the inoculum from the warmed plot (Figure S2).

### 3.2 Results From the Shade House

#### 3.2.1 Shade House Treatment Significantly Altered the Soil Microbiome

**FIGURE 4:**
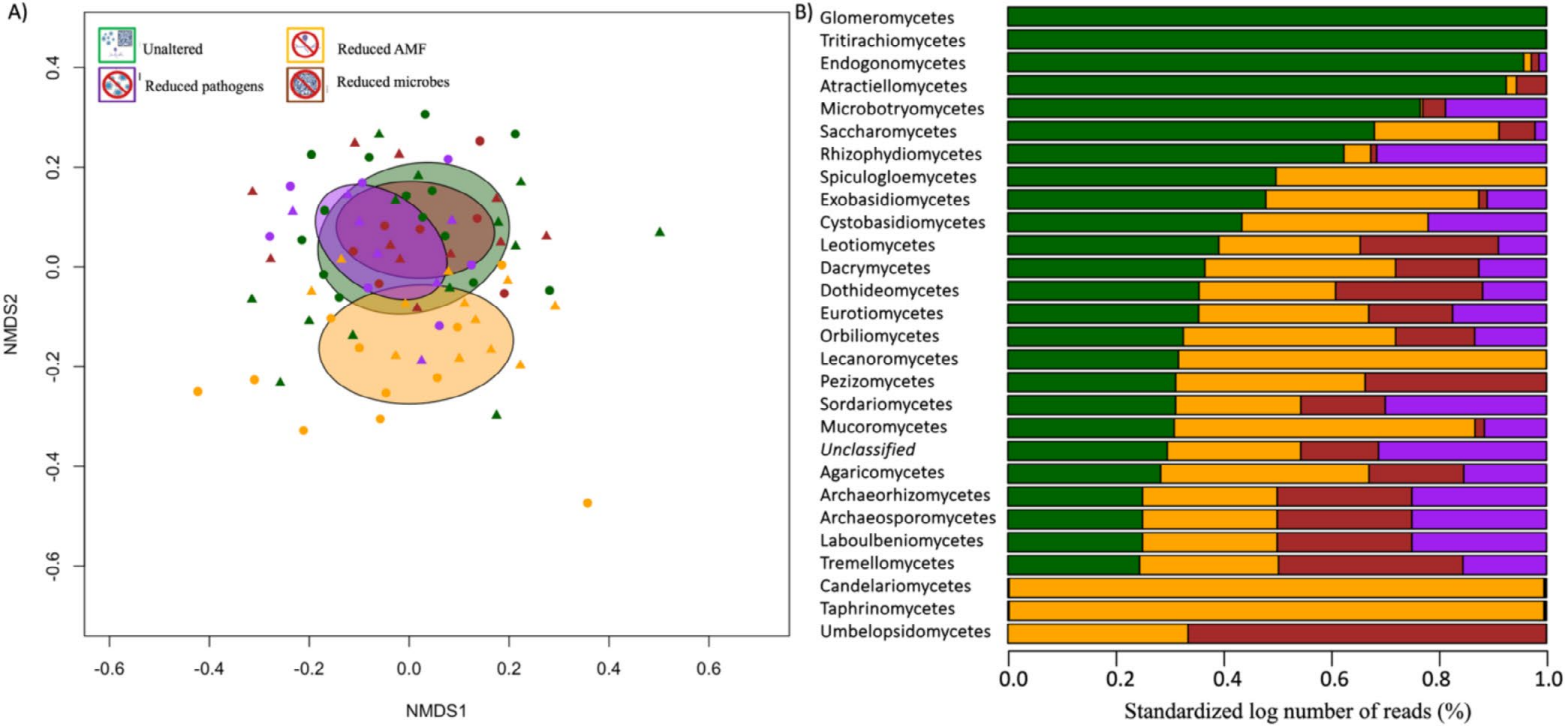
(A) Nonmetric multidimensional scaling to visualize the results of the redundancy analysis showing a significant effect of treatment (color of the symbols) and a treatment by soil source (circle: Ambient plot; triangle: Warmed plot) interaction on fungal composition. (B) Log relative abundance (number of reads), standardized to 1, of fungal classes across the treatments in the shade house experiment, sorted from the highest to the lowest abundant in the unaltered treatment. Reduced arbuscular mycorrhizal fungi (AMF, yellow), reduced fungal pathogens (purple), reduced microbes (brown), unaltered (green).

While the fungal biomass, tested with the linear analysis, remained statistically consistent across treatments in both ambient and warmed plot soil sources (Figure S3), a significant difference in fungal DNA composition was identified across the microbiome treatments (*p* = 0.001, *F*_3,82_ = 1.44, Figure 4A). Additionally, there was a significant interaction between soil source and microbiome treatments (*p* = 0.009, *F*_3,82_ = 1.25). Further analyses across soil sources highlighted key fungal classes altered by the treatments, such as *Agariomycetes* (*p* = 0.07, *F*_3,82_ = 2.40), *Sordariomycetes* (*p* = 0.02, *F*_3,82_ = 3.5), and marginally significant for *Eurotiomycetes* (*p* = 0.15, *F*_3,82_ = 1.81). Post hoc comparisons using the Tukey HSD test showed a significant increase in *Sordariomycetes* relative abundance in the reduced microbes compared with the reduced AMF (difference = 0.59, CI = 0.09: 1.10), and a marginally significant increase in the reduced microbes compared with the reduced pathogens (difference = 0.51, CI = −0.06: 1.08). Nevertheless, we observe a decrease in the log relative abundance of known fungal pathogenic classes such as *Dothideomycetes* in the treatments with reduced fungal pathogens and reduced arbuscular mycorrhizal fungi (AMF), and *Eurotiomycete* and *Sordariomycetes* in the treatments with reduced microbes, reduced fungal pathogens, and reduced AMF, compared to the unaltered treatment. Finally, *Glomeromycetes*, which contains AMF, is only present in the unaltered treatment (Figure 4B).

#### 3.2.2 The Soil Microbiome Significantly Influenced *F*_*V*_ */F_m_* and *T*_*crit*_ in the Shade‐House Experiment

**FIGURE 5:**
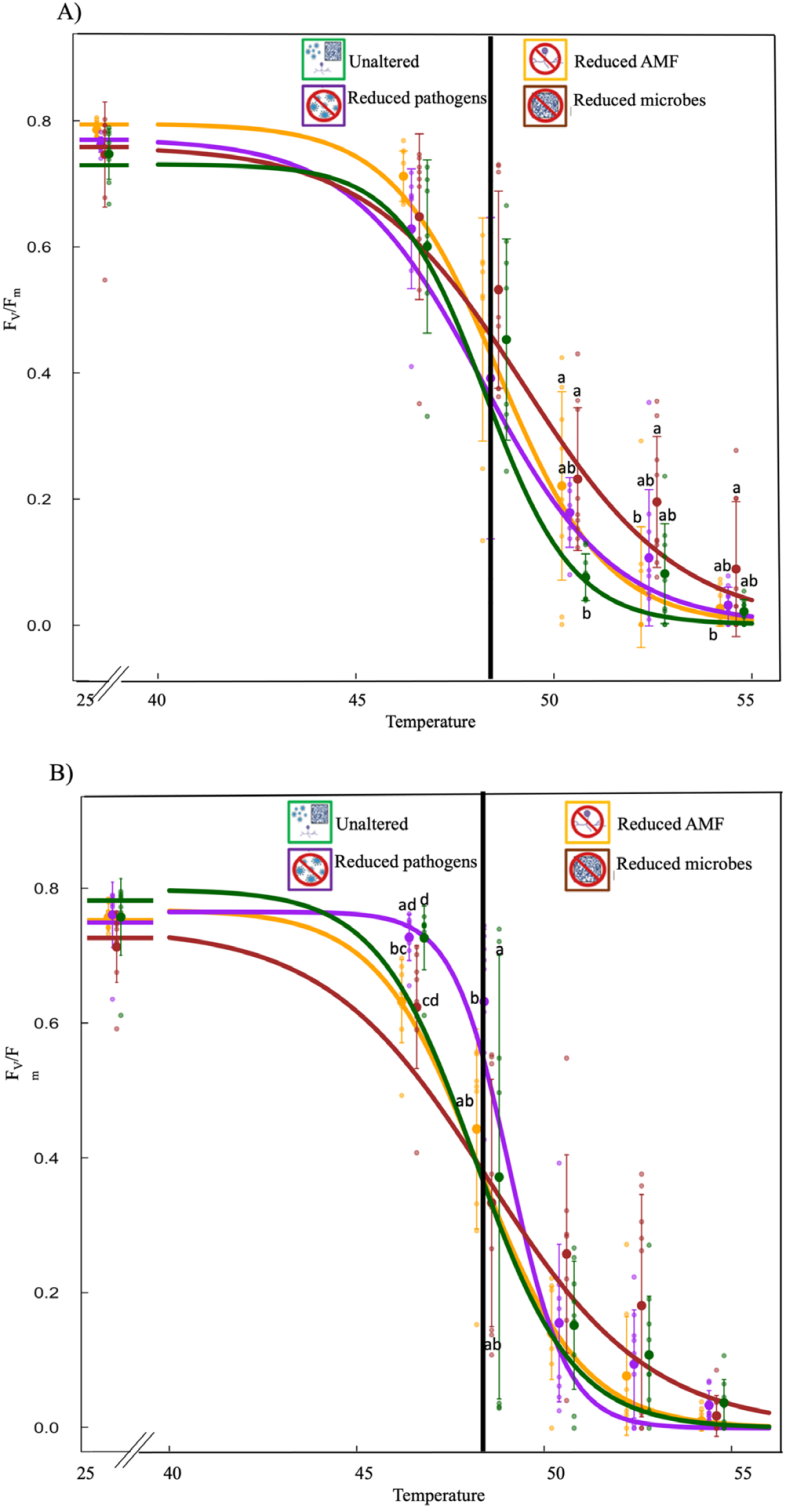
The ratio of maximum variable to maximum total fluorescence (*F*_*V*_/*F*_*m*_) values according to different treatments for ambient temperature (A) and warm (B) soil sources in the shade house. The orange curve is the reduced arbuscular mycorrhizal fungi (AMF) treatment, the purple line is the reduced fungal pathogens treatment, the brown line is the reduced microbes treatment, and the dark green line is the unaltered microbiome treatment. The large dots and the vertical bars indicate the mean and the 95% quantiles of *F*_*V*_/*F*_*m*_ for a given treatment, the small dots are the actual data (*n* = 9 = 3 disks × 3 replicates per temperature). The black vertical line indicates the mean *T*_50_ value across treatments. The letters indicate which pairwise difference was statically significant.

*F*_*V*_ */F*_*m*_ significantly differed across soil microbiome treatments. In the ambient plot soil source, soil microbiome treatment significantly influenced *F*_*V*_ */F*_*m*_ for heat stress at 50°C (*p* = 0.008, *F*_3,44_ = 0.04), 52°C (*p* = 0.031, *F*_3,44_ = 0.03), and 54°C (*p* = 0.072, *F*_3,44_ = 0.01) (Figure 5A, Table S1). Post hoc comparisons using the Tukey HSD test showed plants grown with reduced AMF (difference = 0.14, CI = 0.02: 0.27) and reduced microbes (difference = 0.16, CI = 0.03: 0.28) had significantly higher *F*_*V*_ */F*_*m*_ than plants grown with an unaltered microbiome for a heat stress at 50°C. For heat stress of 52°C (difference = 0.13, CI = 0.01: 0.26), plants grown with reduced microbes showed higher *F*_*V*_ */F*_*m*_ than those grown with reduced AMF.

In the warmed plot soil source, *F*_*V*_ */F*_*m*_ significantly differed across soil microbiome treatments at 46°C (*p* = 0.001, *F*_3,44_ = 0.02) and 48°C (*p* = 0.018, F _3,44_ = 0.14) (Figure 5B, Table S1). Post hoc comparisons using the Tukey HSD test showed the seedlings grown with reduced fungal AMF had lower *F*_*V*_ */F*_*m*_ values than those grown with reduced pathogens (difference = 0.09, CI = 0.01: 0.17) or with an unaltered microbiome (difference = 0.09, CI = 0.01: 0.17) at 46°C. Seedlings grown with unaltered microbiome have significantly higher *F*_*V*_ */F*_*m*_ than seedlings grown with reduced microbes at 46°C (difference = 0.10, CI = 0.02: 0.18). Seedlings grown with reduced fungal pathogens had a significantly higher *F*_*V*_ */F*_*m*_ value than seedlings grown with reduced microbes at both 46°C (difference = 0.10, CI = 0.02: 0.18) and 48°C (difference = 0.29, CI = 0.04: 0.54).

**FIGURE 6:**
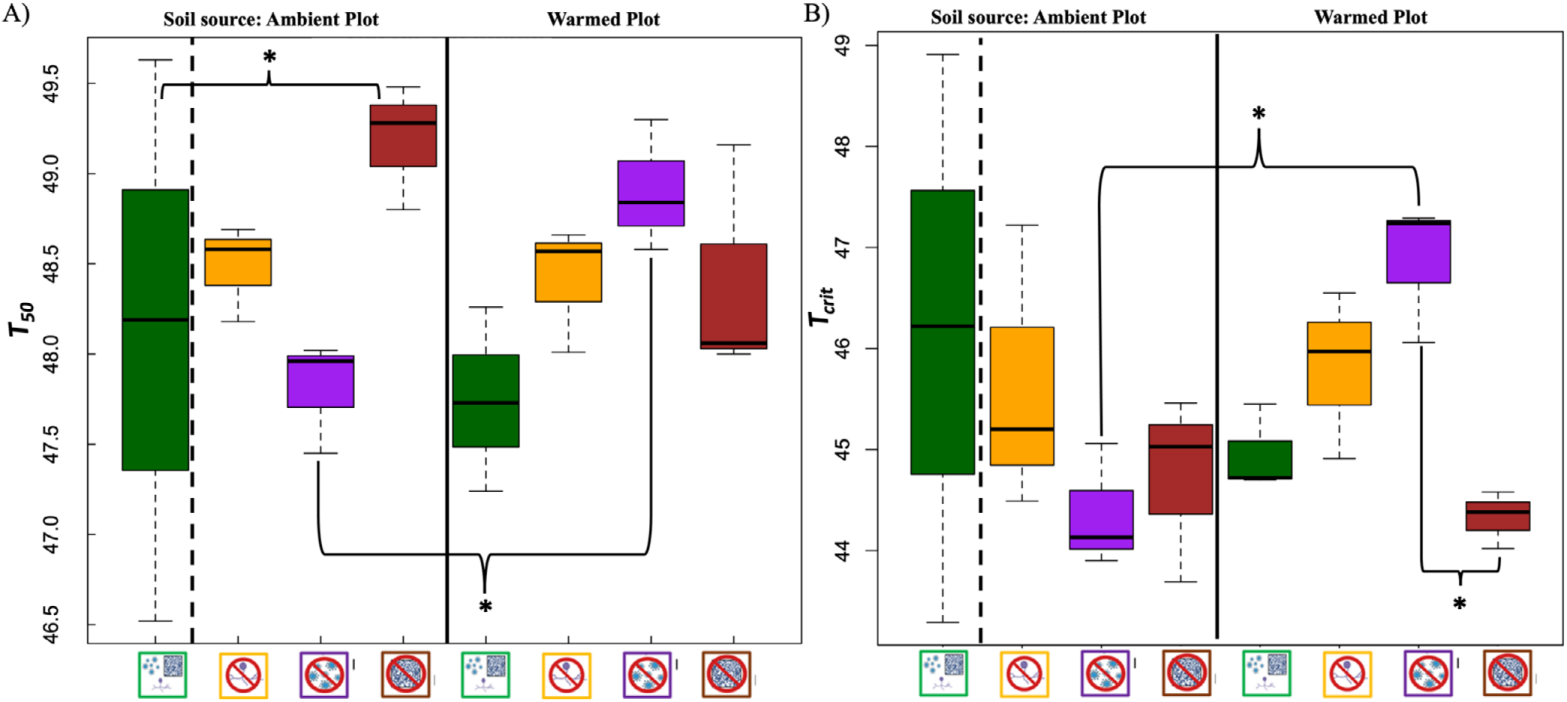
*T*_50_ and *T*_*crit*_ plotted as a function of the interaction between treatment and soil source (*n* = 3 within treatment combinations). Green bar: Unaltered microbiomes; yellow bar: Reduced arbuscular mycorrhizal fungi (AMF); purple bar: Reduced fungal pathogens; brown bar: Reduced microbes. The star indicates a significant difference between pairwise comparisons.

There were significant differences in the two investigated leaf heat tolerance parameters (*T*_50_ and *T*_*crit*_) among the different soil sources and treatments. Post hoc comparisons using the Tukey HSF test showed that *T*_50_ was higher in seedlings from the reduced pathogen treatment from the warmed plot soil source compared to seedlings from the reduced pathogen treatment from the ambient plot soil source (difference = 1.03, SE = 0.44) and significantly higher in the reduced microbe treatment compared with the unaltered treatment in the ambient plot soil inoculum (difference = 1.48, SE = 0.52, Figure 6A). The critical temperature at which heat stress initiates disruption of photosystem II (*T*_*crit*_) was significantly higher in the reduced fungal pathogen treatment (47.50°C) from the warmed plot soil source than in reduced pathogens in the ambient plot soil source (45.5°C) (difference = 2.01, SE = 0.78, Figure 6B). Additionally, *T*_*crit*_ was significantly higher in the reduced microbe treatment compared with the reduced pathogen treatment in warmed plot soil inoculum (difference = 3.19, SE = 0.91, Figure 6B).

## 4 Discussion

Leaf heat tolerance is a trait that provides valuable insight into the capacity of tropical plants to withstand higher temperatures. Using an in situ tropical forest warming experiment, we showed that high temperature impacts might become increasingly detrimental if *Guarea guidonia* experiences high leaf temperature with a reduction in the critical temperature at which heat stress initiates disruption of the photosystem II (Tcri). To investigate further the indirect effects of warming through changes in the soil microbiome, we used a shade house experiment manipulating soil inoculum from the field experiment. Our treatments successfully altered the soil microbiome. As expected, we found an overall negative effect of fungal pathogens on leaf heat tolerance, as seedlings from the reduced fungal pathogens showed a higher *T*_*crit*_ value and *F*_*V*_ */F*_*m*_ values over the other treatments. Additionally, the soil microbiome effects on LHT were inconsistent between soil sources. Our results suggest that the soil microbiome might become increasingly essential to buffer plants against climate change, as the soil microbiome from the warmed plots significantly influenced *F*_*V*_ */F*_*m*_ before *T*_50_, which represents real conditions that plants might experience and be damaged by.

### 4.1 In the Field, Warming Lowers Leaf Heat Tolerance

In the warmed plots, plants exhibited low leaf heat tolerance with a significant reduction in Tcri despite signs of potential acclimation at ambient temperature (25°C). Under 25°C, *F*_*V*_ */F*_*m*_ was significantly higher in plants in the warmed plots compared with the ambient plots. However, this benefit of increasing *F*_*V*_/*F*_*m*_ in the warmed plots disappeared when the leaf disks were incubated at high temperatures (46°C and 52°C). At these temperatures, leaves collected from the ambient plots showed higher *F*_*V*_/*F*_*m*_ values than those collected from the warmed plots. Several mechanisms could explain the drop in *F*_*V*_/*F*_*m*_. First, plants in the warmed plots might have reached their maximal leaf heat tolerance; therefore, any increase in temperature becomes detrimental. Another possible explanation for this result at high temperatures is that plants in the warmed plots also experienced water stress. Prior to the hurricanes, the warming treatment significantly reduced soil moisture at the TRACE site (Kimball et al. 2018); however, soil moisture is more variable across the treatments posthurricane (Cavaleri et al. in prep). Low soil moisture could lower LHT because drought conditions undermine plants' ability to deal with heat by reducing NADP+ concentrations and leading to the breakdown of the oxygen‐evolving complex (Ronde et al. 2004; Alamri et al. 2019; Perdomo et al. 1996). Consistent with this idea, a previous study at our site found that soil moisture better predicted daily physiological responses to heat than vegetation temperature in two understory shrub species (Carter et al. 2020). Since plants in the warmed plots are experiencing a drier environment due to the warming treatment (Kimball et al. 2018), they might respond poorly to the added stress of high temperature and exhibit a decrease in *F*_*V*_/*F*_*m*_.

The result of higher *F*_*V*_/*F*_*m*_ at 25°C in seedlings from the warmed plot compared to seedlings from the ambient plots aligns with studies showing that long‐term acclimation to elevated temperatures increases the LHT (Bäurle 2016; Friedrich et al. 2019; Filaček et al. 2022). Our results are also consistent with previous physiological studies of acclimation at the TRACE site, where shrubs of *Psychotria brachiata* under in situ experimental warming demonstrated the ability to enhance photosynthesis by broadening its thermal niche and increasing its optimum photosynthetic temperature in response to warmer temperatures (Carter et al. 2020). However, at the same site, *Guarea guidonia* did not acclimate to in situ warming (Carter and Cavaleri 2018, Carter et al. 2020; Doughty et al. 2023). Yet, these previous studies at TRACE focused on adult trees (Carter and Cavaleri 2018, Carter et al. 2020; Doughty et al. 2023) whereas we measured leaf heat tolerance on seedlings. Individual plants experience drastic changes in their environment as they mature, which results in leaves with different characteristics in seedlings compared with adult trees (Lawrence et al. 2022). As a result, tolerance to stress, including heat stress, is likely to change during a plant lifespan (Lawrence‐Paul and Lasky 2024). Physiological differences with ontogeny could explain why, at the same sites, we found evidence of acclimation in seedlings, but others found no sign of acclimation at the adult stage in the same species (Carter and Cavaleri 2018, Carter et al. 2020).

Overall, if plants in the warmed plots have a slight advantage under ambient conditions (25°C), further increases in air temperature could have dramatic effects on them in part due to ongoing water stress. Given the climatic predictions that Puerto Rico will experience a warmer and drier climate than it currently is (Henareh et al. 2016), future studies should investigate how tropical forests will respond simultaneously to these stressors.

### 4.2 In the Shade House, the Soil Microbiome Treatments Influenced Fungal Community Composition

In the field, DNA analysis of soil fungi has established that warming treatments have impacted fungal composition. However, the results were only marginally significant, possibly due to our small sample size to avoid large plot disturbances (only one sample per field plot). While fungal classes known for being the most fungal pathogens, such as *Dothideomycetes, Euromycetes*, and *Sordariomycets* (Ohm et al. 2012; Covo 2020; Wang et al. 2023) are similarly present in both inoculants, *Glomeromycetes*, an arbuscular mycorrhizal fungal class, were most prevalent in ambient temperature plots. In contrast, *Archaeosporomycetes*, another arbuscular mycorrhizal fungal class, was only present in the warmed plots. While both classes might show a varied distribution along environmental gradients (Stürmer et al. 2018; Pinto‐Figueroa et al. 2019), *Archaeosporomycetes* abundance may be influenced by both climatic and edaphic factors (Pinto‐Figueroa et al. 2019).

Analyses of soil fungal DNA from the shade house confirmed that the microbiome treatments altered fungal composition. Specifically, in the microbiome treatments with reduced AMF, reduced fungal pathogens, and reduced microbes, the relative abundance of *Eurotiomycetes* and *Sordariomycetes*—classes including many plant pathogens (Morgan and Kamoun 2007; Zhang et al. 2006)—were diminished. Likewise, the *Dothideomycetes* class, which includes plant pathogens that can significantly affect agricultural crops (Haridas et al. 2020), had a smaller relative abundance in the reduced fungal pathogens and reduced AMF treatment. Given the number of unknown fungi at the class level in our soil (21.28%), other fungi important for LHT may be present in our soil even though they have not been identified and linked to LHT yet. Finally, the absence of *Glomeromycetes* in the reduced AMF treatment confirms that the treatment successfully removed the presence of these symbionts. However, the reduced AMF treatment also influenced several fungal pathogens, such as *Dothideomycetes, Eurotiomycetes*, and *Sordariomycetes*. This impact on both beneficial and harmful fungi could explain the lack of a significant effect of this treatment on LHT parameters. Future studies of the impacts of AMF on LHT should consider reducing AMF by preventing root colonization manually using mesh rather than chemically to limit off‐target effects.

### 4.3 In the Shade House, the Soil Microbiome Significantly Influences *T*_50_ and *T*_*crit*_

Consistent with our prediction, reducing fungal pathogens resulted in plants exhibiting a higher heat‐leaf tolerance, suggesting that pathogens negatively affect LHT. Specifically, *T*_50_ and *T*_*crit*_ were significantly higher in plants grown with reduced fungal pathogens in the warmed soil source. Reduced pathogen treatment results in seedlings being better able to deal with heat stress. Plants' vulnerability to diseases may restrict the energy available to sustain a high level of LHT (Matyssek et al. 2005; Smith 2007). Although the mechanisms by which fungal pathogens alter leaf heat tolerance have not been fully elucidated, they may involve intricate interactions between defense responses, signaling pathways, and the ability of plants to adapt to simultaneous or sequential exposure to both heat stress and pathogen attack (Suzuki and Katano 2018). In particular, reduced pathogens might lead to higher resources available for plants to maintain high membrane stability when exposed to high temperature (Sarkar et al. 2018; Rawat et al. 2021; Parasar et al. 2024). Detecting the effect of reduced fungal pathogens on leaf heat tolerance only in a warmed soil source may suggest essential differences between the two soil sources. We detected a significant interaction between soil source and microbiome treatment on the composition of the fungal community. For example, the soil source from the warmed plot inoculum exhibited a smaller relative abundance of potential pathogenic class *Eurotiomyvetes* (Covo 2020). This decrease in fungal pathogens in this soil source and microbiome treatment could potentially explain why the plants exhibited a higher LHT. Fewer pathogens may help the plants create a higher heat tolerance due to the plants' better allocation of resources to deal with heat stress response.

### 4.4 In the Shade House, the Influence of Soil Microbiome on LHT Changes Under Warming Conditions

*F*_*V*_/*F*_*m*_ differed significantly across the soil microbiomes in seedlings grown in ambient plot soil at high temperatures (50°C, 52°C, and at 54°C). Contrary to our expectations, seedlings from the reduced AMF treatment group showed higher *F*_*V*_/*F*_*m*_ than those grown with an unaltered microbiome. These results suggested that Banrot fungicide treatment may have removed more harmful fungi than beneficial mycorrhizal fungi. Soil DNA data does indeed confirm that the reduced AMF treatment in ambient plot soil source removed AMF alongside pathogenic fungi from *Sordariomycetes* and *Dothideomycetes* classes when compared to the unaltered treatment in ambient plot soil source.

*F*_*V*_/*F*_*m*_ was also significantly affected by the soil microbiome from the warmed plot soil source but for heat stress at lower temperatures (46°C and 48°C). As expected, seedlings grown with reduced AMF and reduced microbes had lower *F*_*V*_/*F*_*m*_ (which means a lower tolerance to heat stress) than seedlings grown with an unaltered soil microbiome. This result aligns with previous studies showing that beneficial microbes and fungi, such as mycorrhizal fungi, might enhance the LHT (Hubbard et al. 2014; Shekhawat et al. 2022). Consistent with our predictions, we found that seedlings grown with reduced fungal pathogens had higher *F*_*V*_/*F*_*m*_ than those grown with an unaltered soil microbiome. Therefore, healthier seedlings could better allocate resources to combat heat damage (Chini et al. 2004).

Our study emphasizes the importance of the soil microbiome in buffering plants against climate change. Seedlings from the ambient plot soil source exhibited significant differences in *F*_*V*_/*F*_*m*_ across soil microbiome treatments post‐*T*_50_ mean values. Conversely, significant differences across soil microbiome treatments manifested as pre‐*T*_50_ mean values for seedlings originating from the warmed plot soil source. The *T*_50_ threshold is the temperature at which *F*_*V*_/*F*_*m*_ is reduced by 50% and is an adaptation to extreme leaf temperatures (Perez and Feeley 2020). If the soil microbiome from the warmed plots can modulate *F*_*V*_/*F*_*m*_ post‐*T*_50_, this suggests that the soil microbiome may play an important role in the ability of plants to respond to climate change and potentially further adapt to changing temperature.

The need to investigate how plant–microbiome linkages change under climate change is increasingly recognized (Cao et al. 2020; Nottingham et al. 2022). Previous studies have highlighted how the soil microbiome can help plants tolerate drought (Yang et al. 2009; Ortíz et al. 2015; Ngumbi and Kloepper 2016). Our study provides additional evidence on how the soil microbiome becomes increasingly important for plants to respond to warmer temperatures. Modeling studies have made global predictions suggesting an overall increase in the relative abundance and diversity of fungal plant pathogens (Delgado‐Baquerizo et al. 2020; Li et al. 2023). Our results indicate that pathogens significantly reduced leaf heat tolerance of *G. guidonia* ; therefore, a future where plant fungal pathogens are more abundant and diverse could be associated with more vulnerable plants to extreme leaf temperatures. However, it is important to note that local predictions of increases in fungal plant pathogens abundance and diversity are mixed (Garcia et al. 2020; Morrison et al. 2019), and Puerto Rico is predicted to experience more frequent and intense drought, which could reduce the abundance and diversity of plant fungal pathogens.

## 5 Conclusion

In conclusion, our results highlight the importance of the soil microbiome on leaf heat tolerance in the context of climate change. Consistent with our expectations, two parameters commonly used to characterize leaf heat tolerance, *T*_50_ and *T*_*crit*_, showed significant differences between warmed and ambient plots in the field and/or across microbiome treatments in the shade house. Additionally, differences emerged in *F*_*V*_/*F*_*m*_ values. Notably, the impacts of soil microbiome on leaf heat tolerance were exacerbated in soil from our warmed plots, suggesting that the soil microbiome will become increasingly important with climate change. Our study strongly suggests the need for additional studies on the role of the microbiome in regulating LHT in the context of climate change. We have focused on the soil microbiomes, but future research should include the leaf microbiomes, which directly impact leaf function.

## Author Contributions

**Gabriela Hernandes Villani:** conceptualization (equal), formal analysis (lead), methodology (lead), writing – original draft (lead), writing – review and editing (lead). **Iana F. Grullón‐Penkova:** project administration (equal), resources (equal), supervision (equal). **Parker Bartz:** methodology (equal). **Joel Masanga:** methodology (equal), writing – review and editing (equal). **Jesse R. Lasky:** conceptualization (equal), funding acquisition (equal), writing – review and editing (equal). **Molly A. Cavaleri:** funding acquisition (equal), writing – review and editing (equal). **Tana E. Wood:** funding acquisition (equal), project administration (equal), resources (equal), writing – review and editing (equal). **Benedicte Bachelot:** conceptualization (equal), data curation (lead), formal analysis (equal), funding acquisition (lead), methodology (equal), project administration (equal), supervision (equal), writing – original draft (equal), writing – review and editing (equal).

## Conflicts of Interest

The authors declare no conflicts of interest.

## Supporting information


**Table S1.** Results of the pairwise treatment comparisons in *F*_*V*_ */F*_*m*_ values within soil inoculum source using an analysis of variance (ANOVA) followed by a Tukey post hoc test.
**Figure S1.** Protocol steps: (A) Collect leaf material (Photo of lead author); (B) Punch leaf disks with a paper puncher; (C) Place leaf disks in a cloth and then in Ziploc bags (eggs sinkers used to keep the bag sunk in the water); (D) Apply heat stress in heat‐controlled water baths for 15 min; (E) Place leaf disks on moisture paper in Petri dishes and wait for 24 h; (F) Measure *F*_*V*_ */F*_*m*_ with a chlorophyll fluorometer after dark adapting the leaf disks in the clips for about 15 min.
**Figure S2.** Log Relative abundance (number of reads), standardized to 1, of Fungal Classes in inoculum from the Ambient temperature (blue) or Warmed (red) plots. Dash line is plotted at 50% relative read count.
**Figure S3.** Fungal quantity per treatment in the ambient temperature plot soil source and warmed plot soil source. Green bar: unaltered microbiomes; yellow bar: reduced AMF; purple bar: reduced fungal pathogens; brown bar: reduced microbes.
**Figure S4.** (A) The ratio of maximum variable to maximum total fluorescence (*F*_*V*_ */F*_*m*_) values according to the soil source from the plots (ambient (blue line) and warmed (red line)). The large dots and vertical bars show the *F*_*V*_ */F*_*m*_ mean and 95% quantiles at each temperature. The small dots represent the data points (*n* = 18 = 3 disks × 2 individuals × 3 plots at each temperature for each treatment). The black vertical line represents the mean *T*_50_ of seedlings in ambient and warmed plots. (B, C) The ratio of maximum variable to maximum total fluorescence (*F*_*V*_ */F*_*m*_) values according to different treatments for ambient temperature (B) and warm (C) soil sources in the shade house. The orange curve is the reduced arbuscular mycorrhizal fungi (AMF) treatment, the purple line is the reduced fungal pathogens treatment, the brown line is the reduced microbes treatment, and the dark green line is the unaltered microbiome treatment. The large dots and the vertical bars indicate the mean and the 95% quantiles of *F*_*V*_ */F*_*m*_ for a given treatment, the small dots are the actual data (*n* = 9 = 3 disks × 3 replicates per temperature). The black vertical line indicates the mean *T*_50_ value across treatments. For A–C, the shaded areas represent the confidence intervals of the fitted relationship.

## Data Availability

Final datasets used in the analyses and reproducible code for all results and figures are available on Zenodo: 10.5281/zenodo.14366667.
