# Supplementary material for: Tropical Forest Soil Microbiome Modulates Leaf Heat Tolerance More Strongly Under Warming Than Ambient Conditions
Source: Ecol Evol. 2025 May 14;15(5):e71425. doi: 10.1002/ece3.71425 (PMC12077931; doi:10.1002/ece3.71425)
Supplement: Supplementary file 1 — Table S1. Results of the pairwise treatment comparisons in F V /F m values within soil inoculum source using an analysis of variance (ANOVA) followed by a Tukey post hoc test. Figure S1. Protocol steps: (A) Collect leaf material (Photo of lead author); (B) Punch leaf disks with a paper puncher; (C) Place leaf disks in a cloth and then in Ziploc bags (eggs sinkers used to keep the bag sunk in the water); (D) Apply heat stress in heat‐controlled water baths for 15 min; (E) Place leaf disks on moisture paper in Petri dishes and wait for 24 h; (F) Measure F V /F m with a chlorophyll fluorometer after dark adapting the leaf disks in the clips for about 15 min. Figure S2. Log Relative abundance (number of reads), standardized to 1, of Fungal Classes in inoculum from the Ambient temperature (blue) or Warmed (red) plots. Dash line is plotted at 50% relative read count. Figure S3. Fungal quantity per treatment in the ambient temperature plot soil source and warmed plot soil source. Green bar: unaltered microbiomes; yellow bar: reduced AMF; purple bar: reduced fungal pathogens; brown bar: reduced microbes. Figure S4. (A) The ratio of maximum variable to maximum total fluorescence (F V /F m ) values according to the soil source from the plots (ambient (blue line) and warmed (red line)). The large dots and vertical bars show the F V /F m mean and 95% quantiles at each temperature. The small dots represent the data points (n = 18 = 3 disks × 2 individuals × 3 plots at each temperature for each treatment). The black vertical line represents the mean T 50 of seedlings in ambient and warmed plots. (B, C) The ratio of maximum variable to maximum total fluorescence (F V /F m ) values according to different treatments for ambient temperature (B) and warm (C) soil sources in the shade house. The orange curve is the reduced arbuscular mycorrhizal fungi (AMF) treatment, the purple line is the reduced fungal pathogens treatment, the brown line is the reduced microbes treatment, and the da [file ECE3-15-e71425-s001.docx]

**Supporting Information**

**Table S1.** Results of the pairwise treatment comparisons in *F_V_/F_m_* values within soil inoculum source using an analysis of variance (ANOVA) followed by Tukey post-hoc test.

|  | **Difference** | **Lower** | **Upper** | **P value** | **Temperature** | **Inoculum source** |
| --- | --- | --- | --- | --- | --- | --- |
| Reduced pathogens-Reduced AMF | 0.004 | -0.059 | 0.066 | 0.998 | 25 | Warm |
| Reduced microbes-Reduced AMF | -0.044 | -0.107 | 0.018 | 0.243 | 25 | Warm |
| Unaltered-Reduced AMF | 0.001 | -0.062 | 0.063 | 1.000 | 25 | Warm |
| Reduced microbes-Reduced pathogens | -0.048 | -0.108 | 0.013 | 0.163 | 25 | Warm |
| Unaltered-Reduced pathogens | -0.003 | -0.064 | 0.058 | 0.999 | 25 | Warm |
| Unaltered-Reduced microbes | 0.045 | -0.016 | 0.105 | 0.210 | 25 | Warm |
| **Reduced pathogens-Reduced AMF** | **0.093** | **0.011** | **0.175** | **0.022** | **46** | **Warm** |
| Reduced microbes-Reduced AMF | -0.008 | -0.088 | 0.071 | 0.991 | 46 | Warm |
| **Unaltered-Reduced AMF** | **0.092** | **0.010** | **0.174** | **0.023** | **46** | **Warm** |
| **Reduced microbes-Reduced pathogens** | **-0.101** | **-0.183** | **-0.019** | **0.011** | **46** | **Warm** |
| Unaltered-Reduced pathogens | -0.001 | -0.085 | 0.083 | 1.000 | 46 | Warm |
| **Unaltered-Reduced microbes** | **0.100** | **0.019** | **0.182** | **0.012** | **46** | **Warm** |
| Reduced pathogens-Reduced AMF | 0.185 | -0.064 | 0.434 | 0.205 | 48 | Warm |
| Reduced microbes-Reduced AMF | -0.107 | -0.356 | 0.142 | 0.651 | 48 | Warm |
| Unaltered-Reduced AMF | -0.070 | -0.336 | 0.197 | 0.892 | 48 | Warm |
| **Reduced microbes-Reduced pathogens** | **-0.292** | **-0.541** | **-0.043** | **0.017** | **48** | **Warm** |
| **Unaltered-Reduced pathogens** | **-0.254** | **-0.521** | **0.012** | **0.065** | **48** | **Warm** |
| Unaltered-Reduced microbes | 0.037 | -0.229 | 0.304 | 0.981 | 48 | Warm |
| Reduced pathogens-Reduced AMF | 0.015 | -0.122 | 0.151 | 0.991 | 50 | Warm |
| Reduced microbes-Reduced AMF | 0.115 | -0.026 | 0.256 | 0.143 | 50 | Warm |
| Unaltered-Reduced AMF | 0.011 | -0.125 | 0.148 | 0.996 | 50 | Warm |
| Reduced microbes-Reduced pathogens | 0.100 | -0.041 | 0.241 | 0.238 | 50 | Warm |
| Unaltered-Reduced pathogens | -0.003 | -0.140 | 0.133 | 1.000 | 50 | Warm |
| Unaltered-Reduced microbes | -0.103 | -0.244 | 0.038 | 0.213 | 50 | Warm |
| Reduced pathogens-Reduced AMF | 0.017 | -0.121 | 0.155 | 0.986 | 52 | Warm |
| Reduced microbes-Reduced AMF | 0.102 | -0.036 | 0.240 | 0.208 | 52 | Warm |
| Unaltered-Reduced AMF | 0.030 | -0.108 | 0.168 | 0.932 | 52 | Warm |
| Reduced microbes-Reduced pathogens | 0.085 | -0.053 | 0.223 | 0.361 | 52 | Warm |
| Unaltered-Reduced pathogens | 0.013 | -0.125 | 0.151 | 0.994 | 52 | Warm |
| Unaltered-Reduced microbes | -0.072 | -0.210 | 0.066 | 0.505 | 52 | Warm |
| Reduced pathogens-Reduced AMF | 0.023 | -0.010 | 0.056 | 0.260 | 54 | Warm |
| Reduced microbes-Reduced AMF | 0.007 | -0.026 | 0.040 | 0.933 | 54 | Warm |
| Unaltered-Reduced AMF | 0.026 | -0.007 | 0.059 | 0.158 | 54 | Warm |
| Reduced microbes-Reduced pathogens | -0.016 | -0.049 | 0.017 | 0.583 | 54 | Warm |
| Unaltered-Reduced pathogens | 0.003 | -0.030 | 0.036 | 0.992 | 54 | Warm |
| Unaltered-Reduced microbes | 0.019 | -0.014 | 0.052 | 0.414 | 54 | Warm |
| Reduced pathogens-Reduced AMF | -0.024 | -0.084 | 0.037 | 0.714 | 25 | Control |
| Reduced microbes-Reduced AMF | -0.039 | -0.099 | 0.021 | 0.311 | 25 | Control |
| Unaltered-Reduced AMF | -0.038 | -0.098 | 0.022 | 0.328 | 25 | Control |
| Reduced microbes-Reduced pathogens | -0.015 | -0.076 | 0.045 | 0.898 | 25 | Control |
| Unaltered-Reduced pathogens | -0.015 | -0.075 | 0.045 | 0.911 | 25 | Control |
| Unaltered-Reduced microbes | 0.001 | -0.059 | 0.061 | 1.000 | 25 | Control |
| Reduced pathogens-Reduced AMF | -0.083 | -0.219 | 0.052 | 0.357 | 46 | Control |
| Reduced microbes-Reduced AMF | -0.064 | -0.200 | 0.072 | 0.579 | 46 | Control |
| Unaltered-Reduced AMF | -0.111 | -0.256 | 0.034 | 0.179 | 46 | Control |
| Reduced microbes-Reduced pathogens | 0.019 | -0.117 | 0.155 | 0.981 | 46 | Control |
| Unaltered-Reduced pathogens | -0.028 | -0.173 | 0.117 | 0.951 | 46 | Control |
| Unaltered-Reduced microbes | -0.047 | -0.192 | 0.098 | 0.811 | 46 | Control |
| Reduced pathogens-Reduced AMF | -0.077 | -0.342 | 0.187 | 0.855 | 48 | Control |
| Reduced microbes-Reduced AMF | 0.063 | -0.185 | 0.312 | 0.899 | 48 | Control |
| Unaltered-Reduced AMF | -0.016 | -0.272 | 0.240 | 0.998 | 48 | Control |
| Reduced microbes-Reduced pathogens | 0.140 | -0.117 | 0.398 | 0.458 | 48 | Control |
| Unaltered-Reduced pathogens | 0.061 | -0.203 | 0.326 | 0.920 | 48 | Control |
| Unaltered-Reduced microbes | -0.079 | -0.327 | 0.170 | 0.822 | 48 | Control |
| Reduced pathogens-Reduced AMF | -0.042 | -0.169 | 0.085 | 0.804 | 50 | Control |
| Reduced microbes-Reduced AMF | 0.011 | -0.116 | 0.138 | 0.996 | 50 | Control |
| **Unaltered-Reduced AMF** | **-0.145** | **-0.272** | **-0.018** | **0.020** | **50** | **Control** |
| Reduced microbes-Reduced pathogens | 0.053 | -0.074 | 0.180 | 0.674 | 50 | Control |
| Unaltered-Reduced pathogens | -0.103 | -0.230 | 0.024 | 0.148 | 50 | Control |
| **Unaltered-Reduced microbes** | **-0.156** | **-0.283** | **-0.029** | **0.012** | **50** | **Control** |
| Reduced pathogens-Reduced AMF | 0.046 | -0.078 | 0.171 | 0.743 | 52 | Control |
| **Reduced microbes-Reduced AMF** | **0.135** | **0.011** | **0.259** | **0.029** | **52** | **Control** |
| Unaltered-Reduced AMF | 0.021 | -0.103 | 0.146 | 0.966 | 52 | Control |
| Reduced microbes-Reduced pathogens | 0.088 | -0.036 | 0.213 | 0.236 | 52 | Control |
| Unaltered-Reduced pathogens | -0.025 | -0.149 | 0.099 | 0.947 | 52 | Control |
| Unaltered-Reduced microbes | -0.114 | -0.238 | 0.011 | 0.083 | 52 | Control |
| Reduced pathogens-Reduced AMF | 0.005 | -0.069 | 0.079 | 0.998 | 54 | Control |
| Reduced microbes-Reduced AMF | 0.062 | -0.012 | 0.136 | 0.127 | 54 | Control |
| Unaltered-Reduced AMF | -0.005 | -0.079 | 0.069 | 0.997 | 54 | Control |
| Reduced microbes-Reduced pathogens | 0.057 | -0.017 | 0.131 | 0.175 | 54 | Control |
| Unaltered-Reduced pathogens | -0.010 | -0.084 | 0.064 | 0.983 | 54 | Control |
| **Unaltered-Reduced microbes** | **-0.067** | **-0.141** | **0.007** | **0.085** | **54** | **Control** |


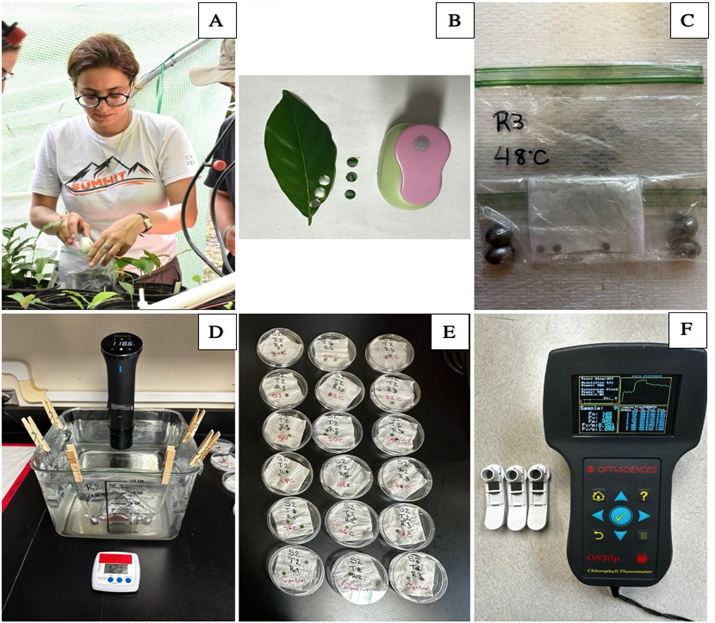


Figure S1: Protocol steps: (A) Collect leaf material (Photo of lead author); (B) Punch leaf disks with a paper puncher; (C) Place leaf disks in a cloth and, then, in Ziploc bags (eggs sinkers used to keep the bag sunk in the water); (D) Apply heat stress in heat controlled water baths for 15 min; (E) Place leaf disks on moisture paper in Petri dishes and wait for 24h; (F) Measure *F_V_/F_m_* with a chlorophyll fluorometer after dark adapt the leaf disks in the clips for about 15 min.

Figure S2: Log Relative abundance (number of reads), standardized to 1, of Fungal Classes in inoculum from the Ambient temperature (blue) or Warmed (red) plots. Dash line is plotted at 50% relative read count.


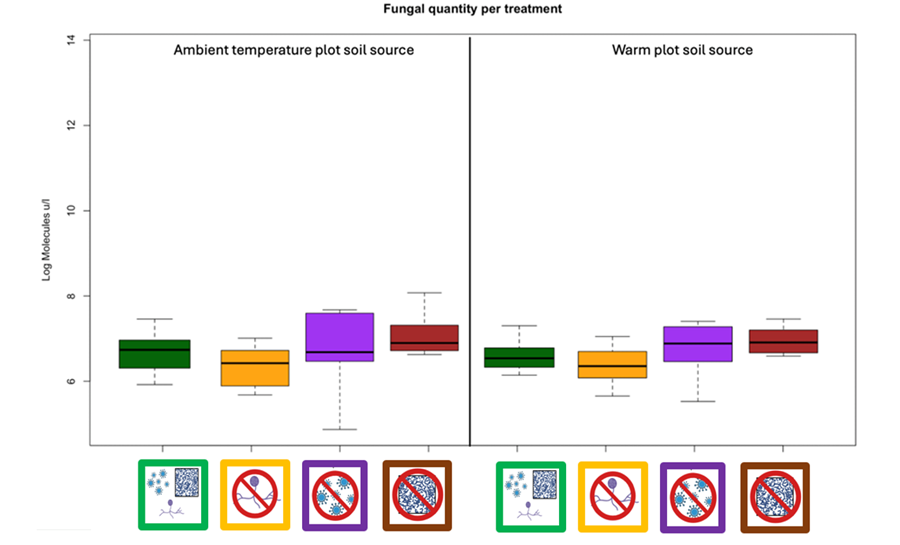


Figure S3: Fungal quantity per treatment in the ambient temperature plot soil source and warmed plot soil source. Green bar: unaltered microbiomes; yellow bar: reduced AMF; purple bar: reduced fungal pathogens; brown bar: reduced microbes.


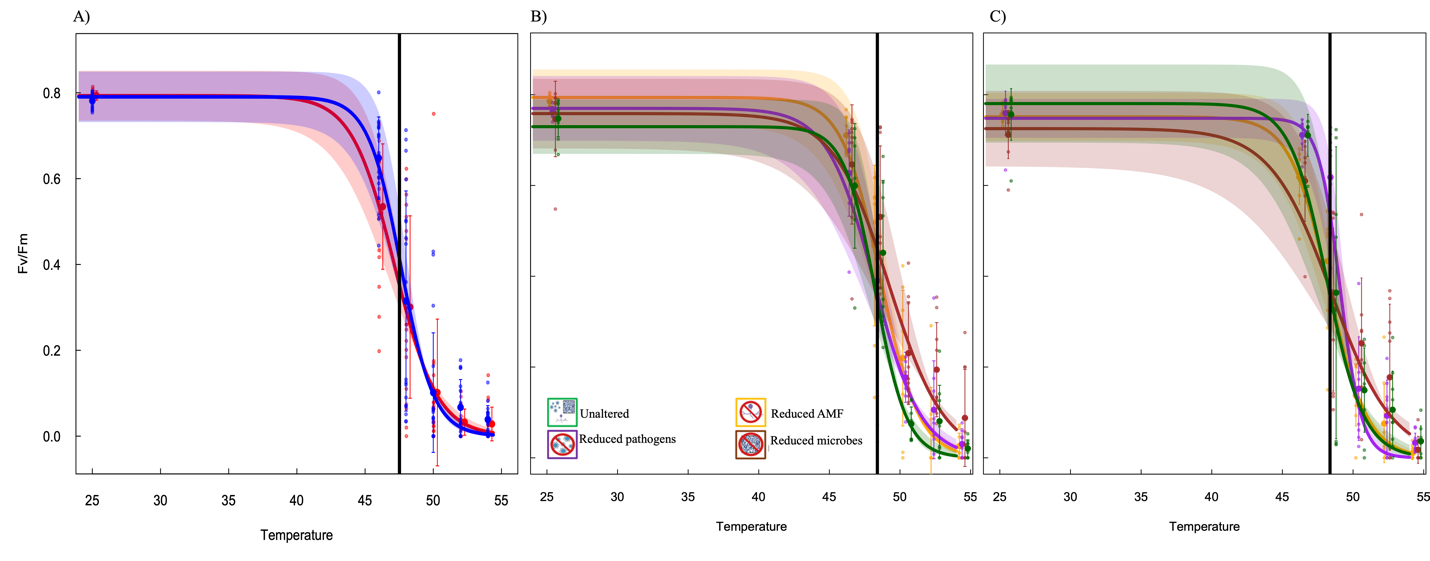


Figure S4: **(**A) The ratio of maximum variable to maximum total fluorescence (*F_V_/F_m_* ) values according to the soil source from the plots (ambient (blue line) and warmed (red line)). The large dots and vertical bars show the *F_V_/F_m_* mean and 95% quantiles at each temperature. The small dots represent the data points (n = 18 = 3 disks x 2 individuals x 3 plots at each temperature for each treatment). The black vertical line represents the mean *T_50_* T50 of seedlings in ambient and warmed plots.B-C) The ratio of maximum variable to maximum total fluorescence (*F_V_/F_m_* ) values according to different treatments for ambient temperature (B) and warm (C) soil sources in the shade house. The orange curve is the reduced arbuscular mycorrhizal fungi (AMF) treatment, the purple line is the reduced fungal pathogens treatment, the brown line is the reduced microbes treatment, and the dark green line is the unaltered microbiome treatment. The large dots and the vertical bars indicate the mean and the 95% quantiles of *F_V_/F_m_* for a given treatment, the small dots are the actual data (n = 9 = 3 disks x 3 replicates per temperature). The black vertical line indicates the mean *T_50_* value across treatments. For A-C, the shaded areas represent the confidence intervals of the fitted relationship.
